# How well do concentric radii approximate population exposure to volcanic hazards?

**DOI:** 10.1007/s00445-023-01686-5

**Published:** 2023-12-19

**Authors:** Sébastien Biass, Susanna F. Jenkins, Josh L. Hayes, George T. Williams, Elinor S. Meredith, Eleanor Tennant, Qingyuan Yang, Geoffrey A. Lerner, Vanesa Burgos, Magfira Syarifuddin, Andrea Verolino

**Affiliations:** 1 Earth Observatory of Singapore, Asian School of the Environment, Nanyang Technological University, Singapore, 639754 Singapore; 2 Department of Earth Sciences, University of Geneva, 13, rue des Maraîchers, CH-1205 Geneva, Switzerland; 3 GNS Science, P.O. Box 30368, Lower Hutt, 5040 New Zealand; 4 Extreme Event Solutions, Verisk, Singapore, Singapore; 5 Earth Observatory of Singapore @ NTU, Interdisciplinary Graduate Programme, Nanyang Technological University, Singapore, 639754 Singapore; 6 Learning the Earth With Artificial Intelligence and Physics (LEAP) National Science Foundation (NSF) Science and Technology Center, Columbia University, New York, NY USA; 7 Geophysical Institute, University of Alaska Fairbanks, Fairbanks, AK USA; 8 State Agriculture Polytechnic of Kupang, Jalan Prof. Herman Yohanes, Kupang, 85228 Indonesia

**Keywords:** Global volcanic exposure analysis, Volcanic hazards, Circular radii, Hazard footprints, Population exposure

## Abstract

Effective risk management requires accurate assessment of population exposure to volcanic hazards. Assessment of this exposure at the large-scale has often relied on circular footprints of various sizes around a volcano to simplify challenges associated with estimating the directionality and distribution of the intensity of volcanic hazards. However, to date, exposure values obtained from circular footprints have never been compared with modelled hazard footprints. Here, we compare hazard and population exposure estimates calculated from concentric radii of 10, 30 and 100 km with those calculated from the simulation of dome- and column-collapse pyroclastic density currents (PDCs), large clasts, and tephra fall across Volcanic Explosivity Index (VEI) 3, 4 and 5 scenarios for 40 volcanoes in Indonesia and the Philippines. We found that a 10 km radius—considered by previous studies to capture hazard footprints and populations exposed for VEI ≤ 3 eruptions—generally overestimates the extent for most simulated hazards, except for column collapse PDCs. A 30 km radius – considered representative of life-threatening VEI ≤ 4 hazards—overestimates the extent of PDCs and large clasts but underestimates the extent of tephra fall. A 100 km radius encapsulates most simulated life-threatening hazards, although there are exceptions for certain combinations of scenario, source parameters, and volcano. In general, we observed a positive correlation between radii- and model-derived population exposure estimates in southeast Asia for all hazards except dome collapse PDC, which is very dependent upon topography. This study shows, for the first time, how and why concentric radii under- or over-estimate hazard extent and population exposure, providing a benchmark for interpreting radii-derived hazard and exposure estimates.

**Supplementary information:**

The online version contains supplementary material available at 10.1007/s00445-023-01686-5.

## Introduction

Accurate exposure assessment is central to risk management and relies upon a spatial representation of the hazard, within which exposed people and/or assets can be quantified. Regional multi-volcano analyses that consider population exposure typically consider hazard footprints as concentric radii extending from 5 km (Ewert and Harpel 2004) to 200 km (Small and Naumann 2001) from an assumed vent location. Concentric radii simplify many challenges encountered in defining hazardous areas around volcanoes, including the identification of eruption source parameters and eruption scenarios based on stratigraphic studies, and their modelling using dedicated tools. Amongst studies using radii-based approaches, the Volcano Population Index (VPI) was developed for Central America by Ewert and Harpel (2004) for radii of 5 and 10 km, with 30 km added for the ranking of US volcanoes by Ewert (2007). This was further expanded to all volcanoes in the Volcanoes of the World Database (VOTW) from the Smithsonian’s Global Volcanism Program (GVP), where the number of people within 5, 10, 30 and 100 km radii is provided as part of the general volcano information (https://volcano.si.edu; Global Volcanism Program 2013). The Population Exposure Index (PEI) of Aspinall et al. (2011) and Brown et al. (2015) subsequently used fatality-weighted population counts within 10, 30 and 100 km radii of volcanoes to rank risk to life from volcanoes. A 10 km radius was considered by all as large enough to capture the hazard footprints and populations exposed for most eruptions (i.e., Volcanic Explosivity Index (VEI) ≤ 3). A 30 km radius was considered by Ewert (2007) to capture proximal populations globally, and to provide a fair representation of exposure to life-threatening hazards accompanying eruptions VEI ≤ 4. A 100 km radius was used in the PEI to capture the majority of life-threatening volcanic hazards for most eruptions, although Brown et al. (2015) recognised that life-threatening hazards from the largest eruptions may extend beyond that. Only one study by Small and Naumann (2001) ranked volcanoes within a global study of population exposure within concentric radii. They identified Gede-Pangrango volcanic complex in Indonesia as the volcano with the highest number of people living within 100 km (29.4 million in 1990). Other global studies using concentric radii have ranked countries for their human population exposure (e.g., Brown et al. 2015; Pan et al. 2015: Indonesia ranked with the highest population), while others have used concentric radii to rank volcanoes within a country or regional level study (Guimarães et al. 2021 and Nieto-Torres et al. 2021, ranking various volcanoes in Latin America using different formulations of the risk equation).

**Fig. 1:**
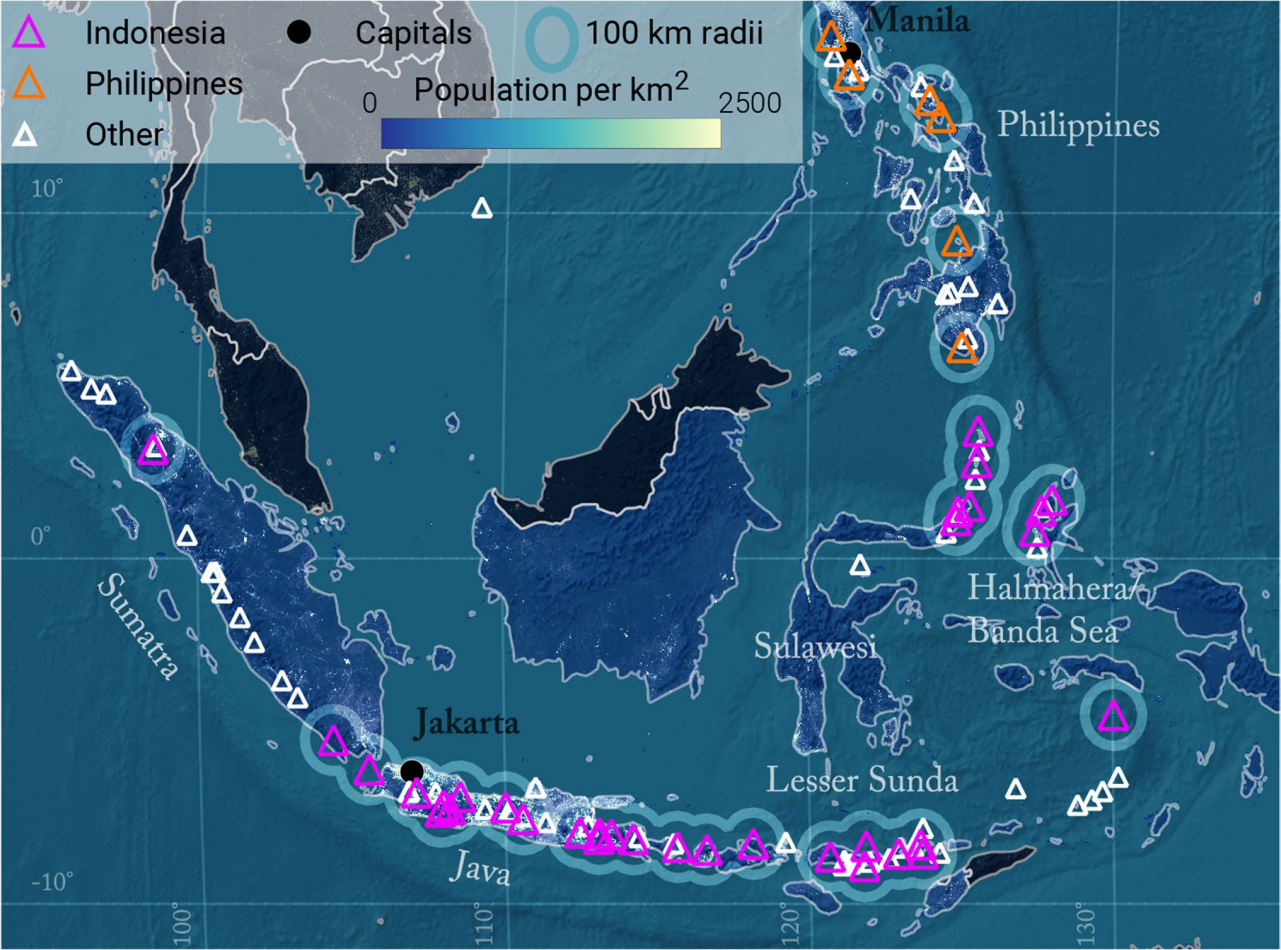
Location of the selected volcanoes in Indonesia (pink) and in the Philippines (orange). White volcanoes are volcanoes in the GVP database that are not considered in the analysis. Gridded population dataset is from LandScan 2018. White labels are regions defined in Jenkins et al. (2022a). The map uses a modified WGS 1984 World Robinson projection centred on the 140^th^ meridian, but radii are estimated using a WGS 1984 Equidistant Conic for Southern Asia projection (ESRI:102029)

Although concentric radii allow for comparison amongst large numbers of volcanoes, they ignore the directionality and change in intensities as a function of distance and direction from the source of most volcanic hazards. Accurate exposure estimates therefore require the spatial relationship between hazard intensity, and the distribution and characteristics of exposed assets to be accounted for. Jenkins et al. (2022a) carried out probabilistic hazard modelling for 40 volcanoes in southeast Asia. Results consistently identified Merapi (Indonesia) as the volcano producing the largest exposure amongst various hazards, which is in contrast with the identification of Gede-Pangrango by Small and Naumann (2001). This observation raises questions regarding the use of radii-based studies. Hence, we investigate here how much exposure calculated from radii differs from model-based analyses that account for the spatial distribution of hazard intensity. To do so, we compare maximum hazard extents and population exposure estimates calculated from concentric radii with those calculated from the simulation of four different volcanic hazards from different eruption scenarios at 40 volcanoes in Indonesia and the Philippines (Fig. 1; study presented in Jenkins et al. 2022a). The 40 volcanoes were chosen based on the occurrence of relatively large explosive eruptions (VEI ≥ 3) and proximity to population. We identify general trends across the volcanoes, and then use three case-study volcanoes—Gede-Pangrango, Cereme, and Merapi in Java—to further investigate why similarities and/or discrepancies exist between population and assets exposure estimates using the two approaches. This provides an evidence-based reference for critically interpreting existing radii-derived estimates of exposure to volcanic hazards.

## Methods

### Hazard modelling

This study relies on the probabilistic simulations of Jenkins et al. (2022a), which assessed the hazard associated with tephra fall loads (using Tephra2; Bonadonna et al. 2005), impact from large clasts (Rossi et al. 2019), and inundation from column (Aravena et al. 2020) and dome (recalibrated version of LaharZ; Schilling 1998; Widiwijayanti et al. 2009) collapse pyroclastic density currents (PDC). For tephra fall, large clast impact, and column collapse PDC, eruptions scenarios of VEI 3, 4 and 5 were considered. In the absence of a relationship between VEI and volume for dome-collapse PDC, Jenkins et al. (2022a, b) modelled volumes 4.5 × 10^5^ m^3^ and 9.8 × 10^6^ m^3^, respectively corresponding to the 50^th^ and 90^th^ percentiles obtained from FlowDat (Ogburn et al. 2016). In addition, Jenkins et al. (2022a) applied two buffers of 300 and 990 m to account for overspill of unconfined PDCs reported in Lerner et al. (2022) for studied eruptions (Merapi 2010; Fuego 2018). In total, 697,080 individual model runs were aggregated into 2,280 scenario-based probabilistic hazard footprints representing conditional exceedance probabilities of 10%, 50% and 90%. For each hazard, exposure of various assets was ranked across the 40 volcanoes. Two separate rankings were developed: a conditional ranking for each VEI or volume and an absolute probability that weighted each eruption scenario by its probability of occurrence to give an overall rank per hazard.

### Exposure calculation

Population exposure was estimated using the 1 km^2^ resolution LandScan 2018 dataset (Rose et al. 2019) in VolcGIS (Biass et al. 2022b; Jenkins et al. 2022a). For tephra fall, we consider exposure to accumulations of 1, 5 and 100 kg/m^2^, which range from disruptive (covering of road markings) to destructive (collapse of the weakest roofs) impacts (Jenkins et al. 2015). For large clast, we consider the maximum distance reached by lapilli resulting in kinetic energies at impact ≥ 30 J as a threshold for skull fracture (Yoganandan et al. 1995). For PDCs, we consider exposure to a binary inundation by the flow reflecting their life-threatening nature.

## Hazard extent

**Fig. 2:**
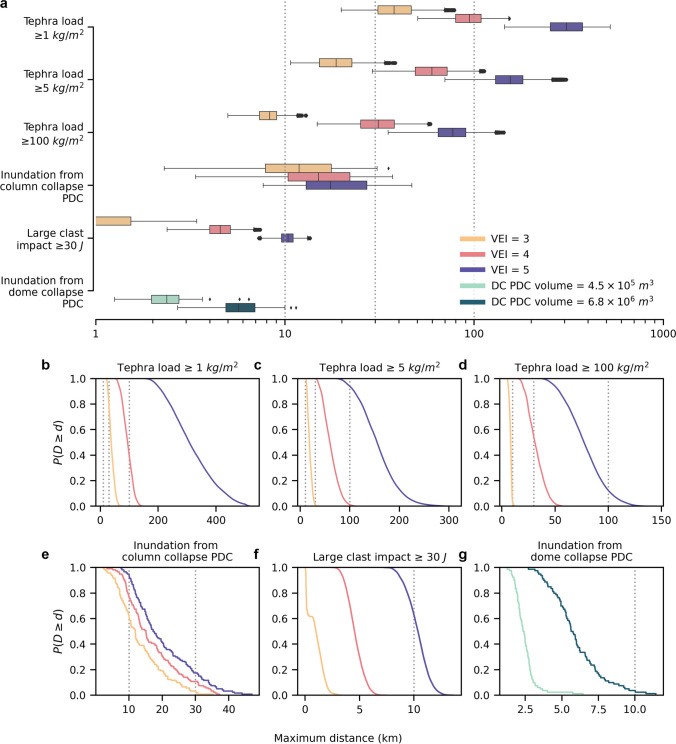
Maximum distance for all hazard footprints across all simulations at the 40 case-study volcanoes. **a** Distances reached by tephra fall loads of 1, 5, and 100 kg/m^2^, inundation from column collapse PDCs, and large clasts impact with kinetic energies ≥ 30 J from VEI 3, 4 and 5 eruptions and for dome collapse PDCs for the two simulated volumes; box edges mark the 25^th^ and 75^th^ percentile and the whisker spans 1.5 × the interquartile range **b–g** Empirical cumulative density functions expressing the probability of a hazard footprint to reach a distance *D* that exceeds a given distance threshold *d.* Radii of 10, 30 and 100 km are shown as dashed black vertical lines

Figure 2 shows the variability in maximum distance reached by the hazard footprints simulated in our study. Tephra fall is the farthest-reaching hazard and the most variable in maximum extent reached. In the present modelling framework (i.e., fixed total grain-size distribution and the use of a time-independent analytical tephra dispersal model), the relationship between plume height and the variability in wind conditions is responsible for the different distances reached by tephra fall for each VEI. By contrast, small-volume dome collapse PDC is generally the most proximal and least variable hazard. The limited reach and variability of dome collapse PDCs reflect the strong control of the H/L parameter and the steep topography of the predominantly stratocone morphology in limiting PDC runout. For column collapse PDC, the selected model outputs inundation exceedance probabilities aggregating thousands of model runs. Since the methodology of Aravena et al. (2020) prevents the access to individual simulations, the spread of distances in Fig. 2 is based on runout distances associated with the 10%, 50% and 90% exceedance probabilities for each modelled VEI and thus does not include the smallest 10% and largest 10% of distances simulated. Overall, Fig. 2 shows that although the 10, 30 and 100 km radii capture the life-threatening hazards for most simulations, the large spread in distances reached reflects the complexity of processes governing volcanic hazards and identifies a discrepancy in exposure estimates from concentric radii.

### How well do concentric radii approximate hazard footprints?

Hazard models variably account for the physical parameterization of volcanic processes as well as non-volcanic factors that influence the spatial distribution of volcanic hazards (topography or wind conditions) and are therefore expected to provide more realistic representations of hazard characteristics than concentric radii. However, a well-informed comparison requires us to review the underpinning rationale for the selection of the radii distances as proxies for hazard footprints.

Based on 191 PDCs, Newhall and Hoblitt (2002) estimated that eruptions of VEI 1–2 and VEI 3 had probabilities of producing PDC runouts exceeding 10 km of 10% and 20%, respectively, although without specifying critical aspects of the considered PDC such as the generation mechanism. This observation was the basis for the choice of a 10 km radius in the Volcano Population Index (VPI) of Ewert and Harpel (2004). By comparison, over 63% of column collapse PDC extents for VEI 3 eruptions and < 4% of dome collapse PDC simulations exceed 10 km (Fig. 2; Table 1). Newhall and Hoblitt (2002) also suggested that eruptions (*n* = 39) of VEI 3 had a 40% probability of producing tephra load accumulations of at least 10 cm (i.e., ~ 100 kg/m^2^) beyond 10 km. By comparison, 3.3% of our VEI 3 tephra simulations exceed the 10 km mark for the 100 kg/m^2^ threshold. None of the smaller simulated volume dome collapse PDCs or VEI 3 large clast simulations extend beyond 10 km.

**Table 1:** Maximum distance reached by all hazards for specific probabilities of occurrence estimated from model simulations. All distance data are provided in Online Resource 1

Distance (km) reached for a given exceedance probability:		5%	10%	25%	50%	75%	90%	95%
Tephra load ≥ kg/m^2^	VEI 3	57.6	53.6	46.5	37.7	31	26.5	24.7
	VEI 4	125.4	118.7	109	94.4	79.9	70	63.7
	VEI 5	461.6	433.5	373.7	307.6	252.8	213.3	195.3
Tephra load ≥ 5 kg/m^2^	VEI 3	27.4	25.4	22.6	18.6	15.2	13.7	13
	VEI 4	89.3	83.1	72.1	59.9	48.8	42	37.8
	VEI 5	224	207.3	181	155	129.8	108.7	96.8
Tephra load ≥ kg/m^2^	VEI 3	9.8	9.5	9	8.3	7.3	6.4	5.9
	VEI 4	46.8	43.6	37.9	31.1	25.2	21.5	19.5
	VEI 5	110.6	102.9	90.4	77.3	64.5	54	48.7
Inundation from column collapse PDC	VEI 3	28.2	24.4	17.6	11.9	7.7	5.9	4.5
	VEI 4	33.9	30.7	22.1	14.7	10.1	8.7	7.2
	VEI 5	36.3	33.9	27.2	17.3	12.9	10.6	9.6
Large clast impact ≥ 30 J	VEI 3	2.1	1.9	1.5	1	0.1	0	0
	VEI 4	6	5.7	5.1	4.5	4	3.6	3.3
	VEI 5	12	11.6	11	10.4	9.6	8.9	8.5
Inundation from dome collapse PDC	4.5e + 05 m^3^	3.6	3.1	2.8	2.4	2	1.7	1.6
	9.8e + 06 m^3^	9.4	8.2	6.9	5.7	4.8	3.8	3.5

The choice of a 30 km radius by Ewert (2007) was similarly based on data from Newhall and Hoblitt (2002), who suggested PDCs from VEI 4–5 eruptions had approximately a 5% chance of exceeding 30 km runout. At a 5% exceedance probability, our simulations suggest a runout distance from column collapse PDC of 34 and 36 km for VEI 4 and VEI 5 eruptions, respectively (Table 1; Fig. 2). For dome collapse PDCs, simulated distances at the 5% exceedance probability are 3.6 km (4.5 × 10^5^ kg) and 9.4 km (9.8 × 10^6^ kg). Newhall and Hoblitt (2002) also indicated a ~ 10% probability of exceeding tephra accumulations of 10 cm at 30 km downwind for VEI 3 eruptions, and 80% for VEI 4 eruptions. Our results suggest that accumulations of 100 kg/m^2^ beyond 30 km occur in 0% and ~ 50% of all simulations for eruptions of VEI 3 and 4, respectively.

A 100 km radius was justified in Brown et al. (2015) as capturing most PDC and lahar flow runouts, while Ewert et al. (2007) did not consider downstream flow hazards (i.e., lahars) to be captured by radii. The volcano fatality database of Brown et al. (2015) indicated that lahar or secondary lahar caused fatal events that typically extended to around 20 km, but with a range of 1–100 km. None of our PDC simulations exceed 50 km (Table 1; Fig. 2) and Jenkins et al. (2022a) did not simulate lahars as their triggering mechanisms and initial conditions cannot be parametrised for such regional studies. For tephra fall, both VEI 4 and 5 eruptions reach beyond 100 km, but only VEI 5 eruptions produce a 12% probability of loads ≥ 100 kg/m^2^ exceeding this distance. Thus, 100 km may be considered as a conservative maximum distance encapsulating PDC and lahar but an underestimate for potentially damaging tephra falls.

## Comparing model- and radii-derived exposure estimates

**Fig. 3:**
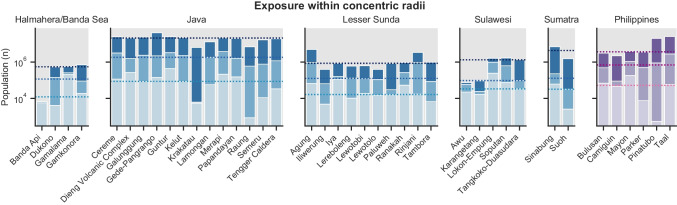
Population exposure (overlying bars) as a function of concentric radii extending 10 (light), 30 (medium) and 100 (dark) km from the vent. Dotted lines mark the median exposure for each radius within each region defined in Fig. 1. Exposure data is available from Jenkins et al. (2022b)

Figure 3 shows population exposure within concentric radii of 10, 30 and 100 km around all 40 volcanoes in our study for the regions shown in Fig. 1. Java and the Philippines dominate exposure within 30 and 100 km, whereas the island volcanoes of Halmahera/Banda Sea and Sulawesi have highest exposure within the 10 km radius. Some volcanoes have relatively low population exposure within 10 km (Raung and Pinatubo) but large exposure within 30 km, while island volcanoes have most of their exposure either concentrated within 10 km (Banda Api, Awu) or between 30 and 100 km away (Krakatau). As found by Small and Naumann (2001), Gede-Pangrango has the largest population exposure within a 100 km radius, which is largely attributed to its proximity to Jakarta, 60 km to the north.

### Comparison with model-derived estimates

**Fig. 4:**
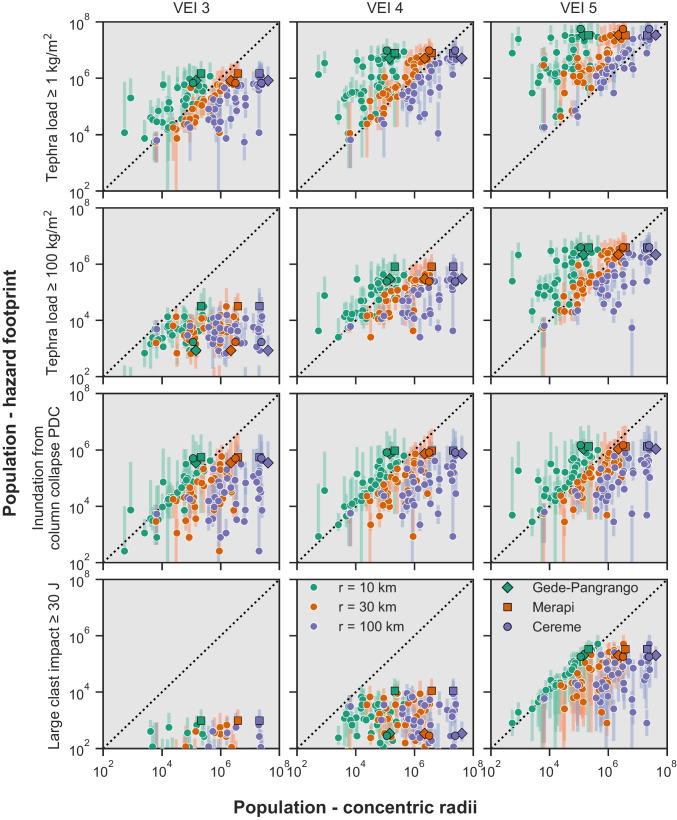
Comparison of the population exposure obtained with the method of concentric radii (x axis) for three different radius sizes versus selected hazard footprints (y axis; 50% probability as dot, 10th to 90th percentile as vertical lines) for three VEI scenarios. The dashed line represents a hypothetical 1:1 fit; points below the dashed line represent larger values from the concentric radii approach, points above the line, larger values from our probabilistic modelling approach. Points with pink, cyan and black edges show Merapi, Cereme and Gede-Pangrango volcanoes, respectively. Gede-Pangrango and Cereme cannot be seen in the lower left plot (VEI 3 large clast impact) because the population exposed is smaller than 100 (*n* = 2). Note that the scale is logarithmic on both axes. Exposure data is available from Jenkins et al. (2022b)

**Fig. 5:**
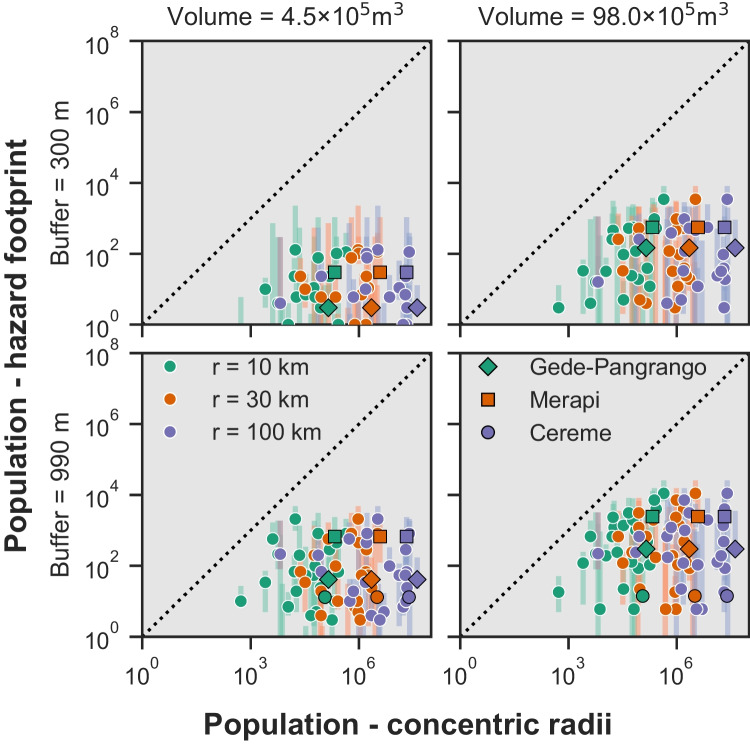
Same as Fig. 4 for dome collapse pyroclastic density current hazard footprints for the two modelled volume scenarios (columns) and two footprint buffers designed to catch overspill and surge (rows). Note that the scale is logarithmic on both axes and different from Fig. 4

Figure 4 compares the populations exposed across the 40 considered volcanoes to concentric radii of 10, 30 and 100 km (x axis) with those exposed to probabilistic footprints of tephra fall accumulations ≥ 1 and ≥ 100 kg/m^2^, column collapse PDC inundation, and large clast impact, for each simulated VEI (3,4,5) (this study: y axis). Figure 5 shows similar data for dome collapse PDC footprints. As a good agreement might be coincidental to the specificity of the region or volcano (i.e., population distribution constrained by the geometry of landforms as a function of the directionality of hazards), we do not suggest updated radii distances that can be used globally. Instead, we use the comparison to highlight by how much exposure estimates can differ as a function of hazard and VEI, and to provide a currently non-existent evidence-based reference for the interpretation of radii-derived exposure analyses. Unless specified otherwise, the following sections discuss a 50% probability of hazard occurrence. A quantitative uncertainty analysis for all probabilities is presented in the Online Resource 2 along with regression analyses for all hazards, probabilities of occurrence and radii.

#### General trends

Results show that, for the most part, concentric radii are conservative and overestimate exposure, although exceptions occur for specific combinations of radii distance, VEI and hazard. Figure 4 shows a general positive relationship between population exposures estimated from concentric radii and modelled tephra fallout, column collapse PDC and large clast impacts footprints. Despite differences in exposure values varying up to multiple orders of magnitude and a large variability amongst hazards and VEI, Fig. 4 suggests that, at a very granular scale, a concentric radii approach can often distinguish high- from low-exposure volcanoes. In contrast, dome collapse PDCs show a poor relationship between exposure estimated from footprints and radii. This can be explained by their typical directionality affecting a limited number of valleys resulting in radii greatly overestimating exposure.

For life-threatening hazards at the volcanoes considered in our study, radii ≥ 30 km (for tephra fallout ≥ 100 kg/m^2^ and column collapse PDC) and ≥ 10 km (for dome collapse PDC and large clasts) typically overestimate population exposure relative to the modelled footprints, particularly for VEI 3 and 4 scenarios. The large clast impact from VEI 5 scenarios shows relatively good alignment with the 10 km radius. For the lower threshold of tephra fall, where impacts may be more disruptive or damaging than directly life-threatening, concentric radii ≥ 30 km (VEI 3 and 4) and ≥ 100 km (VEI 5) appear more aligned with modelled footprints (Fig. 4).

There is significant variation in model- vs radii-derived tephra fall exposure at the volcano scale because of the coincidence, or not, of populations and predominant wind conditions. For example, volcanoes with large conurbations within 100 km but not in the direction of prevailing winds show much reduced exposure when tephra dispersal modelling is employed rather than radii (e.g., Pinatubo, Taal, Gede-Pangrango). Conversely, other volcanoes show increased exposure when wind conditions are considered. This is the case of Cereme, which lies approximately 100 km upwind from Bandung and nearly 200 km upwind from Jakarta, distances that are reached by tephra falls ≥ 1 kg/m^2^ from VEI 4 and VEI 5 eruption, respectively. A similarity in terms of exposed population and trend (i.e., conformance with the 1:1 line) is observed for column collapse PDCs from VEI 3 eruptions with a 10 km radius and from VEI 4 and 5 with radii between 10 and 30 km (Fig. 4). This is a result of modelled column collapse PDCs having an almost circular footprint reaching mostly between ~ 10 and 20 km from source (Fig. 2; Table 1).

#### Case studies

**Fig. 6:**
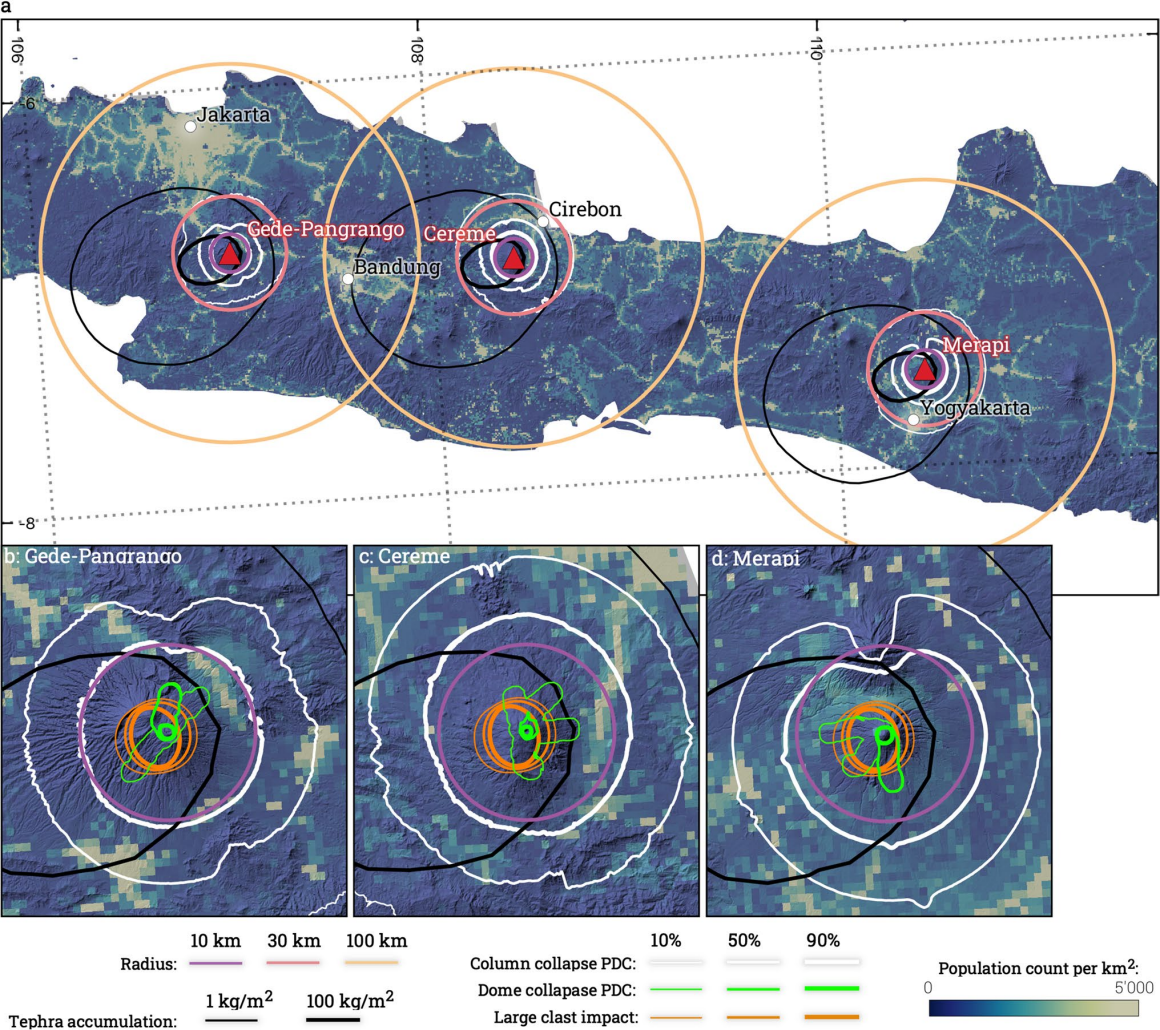
**a** Comparison of population exposure using concentric radii and hazard footprint at Gede-Pangrango, Cereme and Merapi volcanoes on the island of Java (Indonesia). **b**, **c** and **d** zoom on Gede-Pangrango, Cereme and Merapi volcanoes, respectively. Tephra hazard footprints are extracted for a VEI 4 eruption with a 50% conditional probability of occurrence and show the extent of the 1 (thin, black line) and 100 (thick, black line) kg/m^2^. Large clast impact and column collapse PDC inundation are extracted for a VEI 4 eruption and show conditional probabilities of 10% (thin), 50% (medium) and 90% (thick). Dome collapse PDC inundation is extracted for a volume of 9.8 × 10^6^m^3^ and an overspill buffer of 990 m. Topography is the Copernicus GLO-30 dataset (Copernicus DEM, 2023) and population count is Landsat 2018. Maps are projected using a WGS 1984 Equidistant Conic for Southern Asia projection (ESRI:102029) with coordinates shown as EPSG:4326

Using a 100 km radius and 1990 population data, Small and Naumann (2001) identified Gede-Pangrango (Indonesia) as the volcano with the highest population exposure out of 1405 worldwide volcanoes. Across the 40 volcanoes considered in Jenkins et al. (2022a) and using an updated 2018 population data, Gede-Pangrango ranks 8^th^, 7^th^ and 1^st^ for radii of 10, 30 and 100 km, respectively, and illustrates how radii ≥ 30 km progressively include the exposure of Jakarta (10.56 MM people) and Bandung (2.45 MM people; Fig. 6, Table 2). When considering hazard footprints, ranking of the population exposure of Gede-Pangrango across the 40 volcanoes considered in Jenkins et al. (2022a, b) varies between 3 (VEI 5) and 8 (absolute) for tephra accumulations ≥ 1 kg/m^2^ and between 3 (column collapse PDC for VEI ≥ 4) and 30 (large clast impact for VEI 3) for the other modelled hazards (Figures 12–15 of Jenkins et al. 2022a). Figure 6 maps the relative location of the urban centres relative to flow directionality and highlights how the upwind location of Bandung and the crosswind location of Jakarta considerably reduce the total exposure to tephra fallout from Gede-Pangrango when footprints are used. In contrast, Merapi, ranking 7 when considering the exposure within a 100 km circular radius, almost always results in a higher exposure than Gede-Pangrango when modelled footprints are used, and consistently ranks within the five volcanoes with the most exposure to all considered hazards. This is due to the presence of a smaller urban centre (Yogyakarta, 0.42 MM people) within 30 km from the volcano and closer to the main tephra dispersal axis. Finally, Cereme, ranking 4 when using a 100 km buffer, is the volcano with the highest exposure to tephra fallout ≥ 1 kg/m^2^ and PDC inundation for column collapse for VEI ≥ 4, capturing the exposure of Cirebon (0.33 MM people) and Bandung to large eruptions, respectively. It is however interesting to notice that when weighting the exposure from hazard footprints by their long-term probabilities of occurrences (Hayes et al. 2022a), its relatively low eruptive frequency results in Cereme ranking > 10, whereas Merapi ranks 1^st^ for all hazards except inundation from column collapse PDC.

**Table 2:** Summary of exposure for Merapi, Gede-Pangrango and Cereme volcanoes to all hazards and concentric radii in terms of 2018 population count and relative ranks across the 40 volcanoes considered in Jenkins et al. (2022a). All hazards are extracted for a conditional probability of occurrence of 50%. PDC inundation from dome collapse considers an overspill buffer of 990 m. Ranks increase with decreasing exposure. The column **A** represents the rank when the exposure for each VEI is weighted by the median annual probability of occurrence of each VEI scenario obtained using the method of Hayes et al. (2022a) and summed. Since dome collapse PDCs are defined independently from the VEI scale, no long-term absolute exposure is computed

VEI	3			4					5					A		
	Population		Rank	Population		Rank			Population				Rank	Rank		
Population exposed to tephra load ≥ 1 kg/m^2^																
Merapi	1,467,147		1	7,809,433		3			33,456,111				4	1		
Gede-Pangrango	829,163		5	5,098,633		7			33,903,642				3	8		
Cereme	678,746		6	9,620,476		1			56,177,908				1	12		
Population exposed to large clast impacts ≥ 30 J																
Merapi	974		1	110,53		1			333,268				2	3		
Gede-Pangrango	2		30	340		29			201,928				7	7		
Cereme	2		30	284		30			177,819				8	14		
Population exposed to column collapse PDC inundation																
Merapi		557,763	2	951,091		1			1,346,921				2		1	
Gede-Pangrango		350,333	5	741,052		3			1,083,712				3		4	
Cereme		506,865	3	818,166		2			1,482,931				1		11	
Flow volume (m^3^)		4.5 × 10^5^		9.8 × 10^6^												
		Population	Rank	Population		Rank										**–**
Population exposed to dome collapse PDC inundation																
Merapi		668	4	2,453		5										–
Gede-Pangrango		41	15	297		14										–
Cereme		13	19	14		25										–
Radius (km)		10		30				100								
		Population	Rank	Population		Rank		Population		Rank						
Population within concentric radius																
Merapi	213,561		4		3,845,569	1	20,912,606				7					–
Gede-Pangrango	142,252		8		2,220,611	7	41,052,844				1					–
Cereme	114,326		9		3,181,418	3	24,363,615				4					–

## Conclusions and future directions

Estimating volcanic hazards as concentric radii facilitates comparison across multiple volcanoes and is therefore a popular approach in regional or global assessments. This study provides the first benchmark between this radii approach and the use of models for volcanic hazard assessments. A critical interpretation of the distances reported in Fig. 2 requires understanding the conceptual foundations for radii-based versus modelled-based hazard footprints. In most studies (e.g., Brown et al. 2015; Ewert 2007; Ewert and Harpel 2004), radii are identified based on a compilation of past events by Newhall and Hoblitt (2002). Although representing actual realisations of natural events, their limited witnessed occurrences might not represent the full scope of possibilities that could occur in future eruptions (Bonadonna 2006). Inferring a circular hazard footprint from maximum runouts of observed processes intrinsically implies an equal radial probability of occurrence, an assumption that was not made by Newhall and Hoblitt (2002) who considered directionality in the development of event-trees. In contrast, modelling footprints rely on approximations of natural processes based on a range of numerical, analytical and empirical techniques which, when combined with probabilistic modelling methodologies, allow for the exploration of possible outcomes that have not necessarily yet been realised. Regardless of the nature of the model used, modelled footprints better reproduce the directionality of hazards but are always more demanding in terms of computing power and parametrisation (number of eruption source parameters and other input conditions). Global hazard modelling is now becoming viable thanks to the increasing available computing power and dedicated open-source software (e.g., Bertin et al. 2019; Biass et al. 2016; Mahmood et al. 2015; Palma et al. 2014; Tierz et al. 2017), potentially opening the door to global Probabilistic Volcanic Hazard Assessments (PVHA). However, such regional to global studies require a balance between model sophistication and the computing power and input data available.

Regarding exposure analysis, results show that except for tephra, population estimates are typically larger using radii than modelled footprints. Although some relationships between the radii-derived and model-derived exposure estimates might appear similar on log-log plots of Figs. 4 and 6, the quantitative error analysis presented in Online Resource 2 reveals a scatter that can span orders of magnitude. In addition, an agreement between both methods can be coincidental due to the distribution of inhabited areas. Consequently, we deliberately restrict the scope of our study to a direct comparison between the results of both approaches rather than overly interpreting any relationships between or within the results, and our study only intends to provide an evidence-based reference for critically interpreting existing radii-derived estimates of exposure to volcanic hazards. Should the study be used to guide future applications of radii to exposure assessment, the findings must be evaluated considering the geographic scope of our study and the magnitude of errors, i.e. the confidence intervals from Figs. 4 and 5.

In conclusion, our study provides a benchmark for objectively comparing hazard analyses based on concentric radii and modelled hazard footprints (Fig. 2; Table 1) and reveals that:

- A radius of 10 km generally underestimates the extent of VEI 3 hazard footprints for column collapse PDC and overestimates the extent for tephra loads ≥ 100 kg/m^2^, large clasts impacts ≥ 30 J and dome collapse PDC.
- A radius of 30 km generally underestimates the footprint extent of tephra fallout for VEI ≥ 4 and represents a median value of distances reached by a 100 kg/m^2^ load from a VEI 4 eruption. A 30 km radius generally overestimates the runout of column collapse PDCs (e.g., VEI 4 and 5 eruptions have only a ~ 5% exceedance probability to exceed 34 and 36 km, respectively).
- Only tephra fall from VEI 4 and 5 eruptions are likely to exceed distances of 100 km. VEI 5 eruptions have a 12% probability to produce tephra loads ≥ 100 kg/m^2^ beyond 100 km.

Regarding population exposure in southeast Asia, our analysis suggests that:

- There is a general positive relationship between population exposure derived from radii and from hazard footprints for tephra fallout, column collapse PDC and large clast impacts, but not for dome collapse PDC, which is very strongly dependent upon local topography.
- The selected radii i) dominantly overestimate population exposure to column collapse PDC, ii) almost exclusively overestimate population exposure to large clast impacts and iii) always overestimate population exposure to dome collapse PDC. Only population exposure to tephra fallout can be underestimated by the radii approach depending on the load threshold and the VEI.
- These observations must be analysed in the perspective of the large (i.e., orders of magnitude) errors and the potential coincidental population distribution within concentric radii and directional hazard footprints.

In addition to the development of global hazard modelling methodologies, we identify three future research directions for global volcanic hazard, exposure and impact assessment:

- The development of global PVHA that accounts for the spatiotemporal probability of eruptions (e.g., Deligne et al. 2010; Hayes et al. 2022a; Rougier et al. 2018; Sheldrake et al. 2020) and systematically estimate uncertainties (Marzocchi et al. 2010).
- Exposure analyses that consider more assets than only population (Biass et al. 2017; Hayes et al. 2022b), which is made possible by crowdsourcing, modern spatial data infrastructures and machine learning applied to big Earth Observation data (Biass et al. 2022a, b; Buchhorn et al. 2020; Giuliani et al. 2019; Gorelick et al. 2017).
- The development of methodologies that estimate the potential consequences on the exposed populations and assets. This requires the parametrisation of vulnerability, which is commonly achieved using a combination of opportunistic post-event impact assessments (e.g., Elissondo et al. 2016; Jenkins et al. 2017; Magill et al. 2013), experiments (e.g., Ligot et al. 2023; Wardman et al. 2012; Williams et al. 2021) and theoretical studies (Jenkins et al. 2014). Until now, the majority of studies to date have concentrated on physical impacts from tephra fall and to buildings (Deligne et al. 2022). New efforts must attempt capturing direct impact on other assets as well as other dimensions of vulnerability relevant to risk reduction actions (socio-economic impacts, systemic vulnerability).

### Supplementary information

Below is the link to the electronic supplementary material.

Supplementary file1 (DOCX 15 KB) Online resource 1: Maximum hazard footprint distance data from simulations available at https://doi.org/10.21979/N9/GWNX5K.

Supplementary file2 (XLSX 19 KB) Online resource 2: Error analysis for population exposure available at https://doi.org/10.21979/N9/1Z99TC.
