# Supplementary material for: How well do concentric radii approximate population exposure to volcanic hazards?
Source: Bull Volcanol. 2023 Dec 19;86(1):3. doi: 10.1007/s00445-023-01686-5 (PMC10730679; doi:10.1007/s00445-023-01686-5)
Supplement: Supplementary file 1 — Supplementary file1 (DOCX 15 KB) Online resource 1: Maximum hazard footprint distance data from simulations available at https://doi.org/10.21979/N9/GWNX5K. [file 445_2023_1686_MOESM1_ESM.docx]

**Online resources to manuscript: How well do concentric radii approximate population exposure to volcanic hazards?**

Biass et al.

**Online resource 1**

Online resource 1 contains the maximum distances reached by hazard footprints from our simulations used to reproduce Figure 2 and Table 1 of the manuscript. The zip file contains one .csv file for each hazard containing a column maxD representing the maximum hazard extent in metres.

**Online resource 3**

Online resource 3 compares the population exposures calculated from modelling with those calculated from radii in terms of their mean percentage error (MPE) and Chi-square error, which are two order-dependent error functions. This gives us information about the dispersion around the 1:1 modelled:radii line. The MPE provides a proportional deviation from the model’s mean prediction but results in larger penalties for overestimations. For instance, negative MPE values (i.e., when the exposure of footprints is lower than buffers) are bounded to -100%, whereas positive MPE values have no upper limit. In contrast, the Chi-square provides a more symmetrical error.
